# The impact of track and field training on dynapenia: gender differences in age-related decline of vertical jump performance among older adults

**DOI:** 10.3389/fragi.2024.1504789

**Published:** 2024-12-12

**Authors:** Eneko Fernández-Peña, Eugenio Formiglio, Marco Gervasi, Piero Benelli, Alexander Bertuccioli, Giuseppe Russo, Valerio Giustino, Antonino Patti

**Affiliations:** ^1^ Department of Physical Education and Sport, University of the Basque Country UPV/EHU, Vitoria-Gasteiz, Spain; ^2^ Department of Biomolecular Sciences, University of Urbino Carlo Bo, Urbino, Italy; ^3^ Sport and Exercise Sciences Research Unit, Department of Psychology, Educational Science and Human Movement, University of Palermo, Palermo, Italy

**Keywords:** aging, countermovement jump, sporting activity, gender, inactive elderly, lower limb strength

## Abstract

**Introduction:**

Alongside sarcopenia, the age-related loss of muscle strength and power, known as dynapenia, increases the risk of functional disability and mortality in older adults. However, engaging in sporting activities during old age appears to enhance functional capacity. The differences in effects between athletes and sedentary individuals, as well as between genders, have yet to be fully clarified.

**Methods:**

The vertical jump test is recognized as a measure of lower limb performance with almost no learning effect. In the present study, we quantified age-related countermovement jump (CMJ) height loss in 120 subjects over 58 years old among both master athletes and sedentary counterparts, and analysed gender differences.

**Results:**

Both male and female master athletes showed significantly higher jump heights results than their sedentary counterparts (male athletes 28.5 ± 4.3 cm vs. male sedentaries 15.1 ± 5.2 cm; *p* < 0.01; female athletes 22.7 ± 2.5 cm vs. female sedentaries 8.2 ± 3.3 cm; *p* < 0.01). Female athletes were found to have higher CMJ performance than even sedentary men (*p* < 0.01). The rate of decline in jumping ability was the same for male athletes and non-athletes, but female athletes had the shallower rate of decline of all the groups observed (2.78 cm per decade).

**Discussion:**

Sporting activity in the older age allows both men and women to perform at a higher level, with the latter also benefiting from a slower rate of decline, which can have a positive impact on functional ability and quality of life.

## 1 Introduction

The world’s elderly population continues to grow at an unprecedented rate. In almost all countries, the proportion of people over 60 is growing faster than in any other age group, due to both longer life expectancy and declining fertility rates (U.S. Census Bureau, 2020). According to the latest World Health Organization report on aging and health, there is little evidence that increasing longevity is accompanied by a long period of good health (Beard et al., 2016). Frailty is a syndrome related to the aging of several physiological systems, resulting in a situation of vulnerability for older people. The etiology of frailty is influenced by the interaction of numerous factors like fatigue, polypharmacy, weight loss and inadequate nutrition (Morley, 2016), with sarcopenia emerging as the primary contributor. This condition is strongly associated with increased adverse outcomes including falls, functional decline, frailty and mortality (Cruz-Jentoft and Sayer, 2019). In addition to sarcopenia, older persons are more vulnerable to functional limitations and death due to age-related decrease of muscular strength and power, or dynapenia (Clark and Manini, 2012). These functional limitations of the lower limbs include even the most common tasks of daily life such as incline walking, climbing stairs (Grimmer et al., 2019) or getting up from a chair (Alexander et al., 1991).

Recent studies confirm that function-related loss of strength in aging is a factor most closely linked to the loss of motor units’ recruitment and the loss of the rate of motor unit firings, i.e., the ability to exert muscle power. In this regard, sporting activities at older age can reduce the comorbidity effect of aging (Gervasi et al., 2017; Agostini et al., 2023). The vertical jump test has gained recognition over the years as an intuitive measure of lower limb power with almost no learning effect (Rittweger et al., 2004) and strong ecological validity (Hong et al., 2021). The full chain of anti-gravity muscles needed for numerous regular physiological tasks is assessed, and the maximal power and height of the jump generated during a countermovement jump (CMJ) can be calculated. The aging decline in vertical jump power has already been seen in a sample of non-athletes (Siglinsky et al., 2015) and in master athletes (Michaelis et al., 2008; Alvero-Cruz et al., 2021). However, to the best of our knowledge, no study has compared athletes and sedentaries to assess the influence of sporting activity and gender in CMJ height in elderly people. Therefore, the objectives of this observational study were: (i) to measure the decline in CMJ height in both master athletes and sedentary older individuals; and (ii) to assess the effect of physical activity and gender on the decline in CMJ height of older people.

## 2 Materials and methods

### 2.1 Participants

Sixty-four italian track and field master athletes participating at the European Masters Athletics Festival for Silver Age (EMAF) were recruited to participate in the study, as well as fifty-six healthy sedentary volunteers. The characteristics of both groups are reported in Table 1. The inclusion criteria required participants to be between 55 and 90 years old and in good health at the time of the study. Specifically, participants needed to maintain daily functional abilities such as walking and engaging in household activities, and they should not have acute or chronic conditions (such as heart disease or diabetes) (World Health Organization, 2015). For athletes, a minimum of 3 years of continuous training in track and field prior to the study was required. Most participants competed in multiple track and field events, including endurance running (800–10,000 m), sprinting (60–400 m, with and without hurdles), as well as throwing and jumping disciplines. They did not engage in sports outside of track and field. Exclusion criteria included smoking, consuming more than three alcoholic drinks per day, or undergoing treatment with drugs that affect muscle recovery and musculoskeletal performance. All participants provided written informed consent to participate in the study, following a medical health screening. The protocol was approved by the Ethics Committee of the University of Urbino “Carlo Bo”, Italy (No. 31_2020) and was conducted in accordance with the Declaration of Helsinki for research with human volunteers. All participants were assessed for height and weight and the Body Mass Index was calculated. Before starting the experimental phase, all participants familiarized with the warm-up and CMJs.

**TABLE 1:** Demographic and anthropometric characteristics of participants.

Groups	N	Age (years)	Weight (kg)	Height (cm)	BMI (kg/m^2^)
Male athletes	39	66.7 ± 6.7	72.2 ± 10.7	176.3 ± 7.5	23.1 ± 2.4
Female athletes	25	70.7 ± 7.5	61.4 ± 6.0	168.5 ± 6.9	21.7 ± 2.0
Male sedentary	29	70.3 ± 6.4	72.8 ± 7.8	168.2 ± 5.5	25.8 ± 3.3
Female sedentary	27	69.1 ± 6.2	63.0 ± 9.6	157.6 ± 6.6	25.4 ± 3.2

### 2.2 Familiarization

**FIGURE 1:**
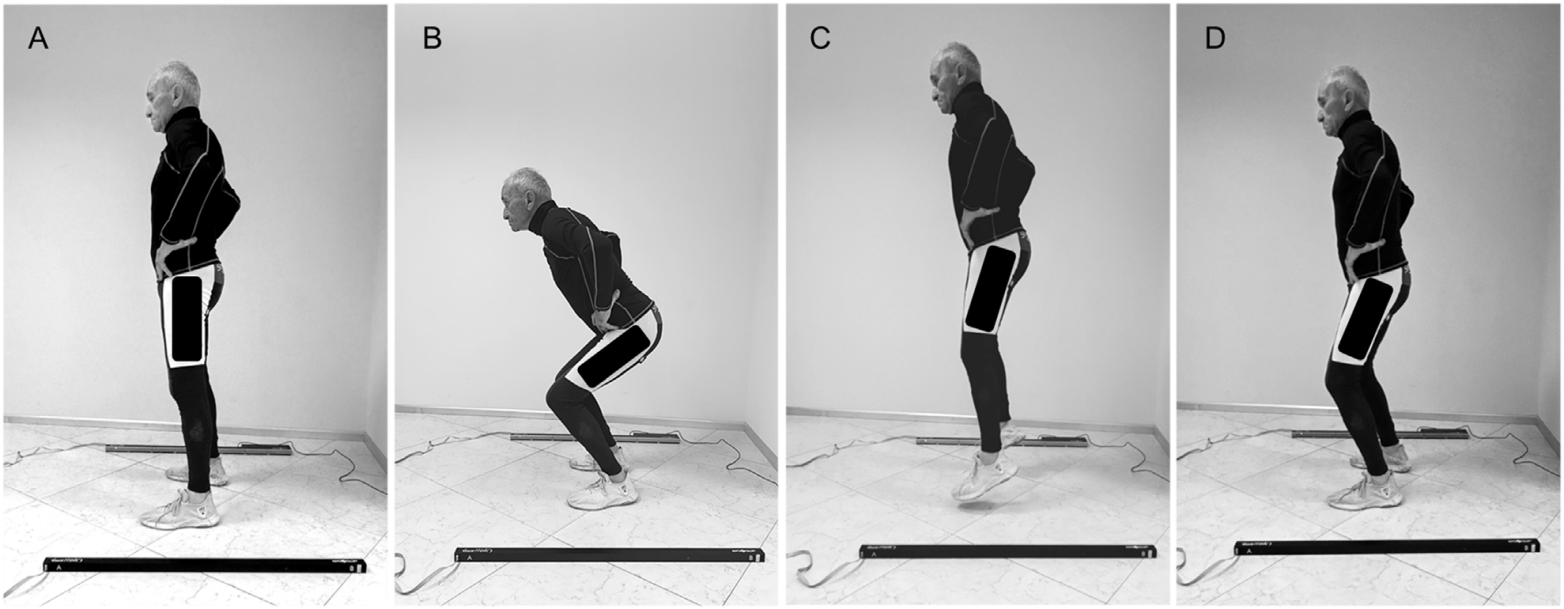
A sequence of an 84-year-old male participant performing a CMJ. **(A)** Initial Standing Phase; **(B)** Braking Phase; **(C)** Flight Phase; and **(D)** Landing Phase.

To avoid injuries in the sedentary participants, they performed six 30-minute familiarization sessions. Each session consisted of a speed walk or light jog alternating with recovery breaks until at least 4-minute continuous jogging were reached. In addition, all participants were taught some basic running drills for specific activation of the muscles involved in vertical jumping (quadriceps, biceps femoris, gluteus and gastrocnemii). Finally, at the end of the last three of familiarization sessions, the participants performed six CMJs under the supervision of two experts in physical exercise who instructed them to jump with a knee angle of approximately 90°. In the only six cases (≈10.7%) where sedentary participants could not meet this criterion, smaller knee flexions (around 100°) were accepted as long as the jumps were performed maximally (Figure 1).

For the Master athletes, two familiarization sessions were conducted on the 2 days prior to the experimental protocol. These sessions included a warm-up phase similar to that of the sedentary athletes, followed by six CMJs performed with proper technique: hands on hips, feet hip-width apart, starting with an upright torso, a quick bend of the knees to approximately 90°, and a jump initiated by the rapid extension of the lower limbs.

### 2.3 Experimental procedures

On the day following the final familiarization session, all participants completed the experimental protocol. This began with a 15-minute warm-up, consisting of 5 min of walking or running, followed by 5 min of dynamic mobility exercises for the upper and lower body. At the end of the warm-up, participants performed four specific running drills: Skips (to activate glutes and hamstrings), High Knees (running in place or moving forward while lifting the knees as high as possible), Butt Kicks (running in place or moving forward while kicking the heels up to touch the glutes), and Ankling (focusing on quick, short steps with stiff ankles). After a 3-minute recovery period, participants performed three CMJs with 1 min of rest between each jump. Participants were encouraged to exert maximal effort for each jump. Jump height was measured using the Optojump (Microgate SrL) system, which calculates jump height indirectly based on flight time. The height of the highest of the three jumps was used for data analysis.

### 2.4 Statistical analysis

Linear regression analysis was used to assess the correlation between age and jump height, and a test for the equality of slopes was used to compare the slopes of two independent samples (athletes vs sedentaries, or males vs females). An ANCOVA test was performed using JASP (version 0.19.1) to compare the means of different groups, with gender and physical activity as factors and age as covariate. A *post hoc* with Bonferroni correction was used to detect significant differences between groups. The critical level of significance for the statistical tests was set at 0.05 (5%).

## 3 Results

**FIGURE 2:**
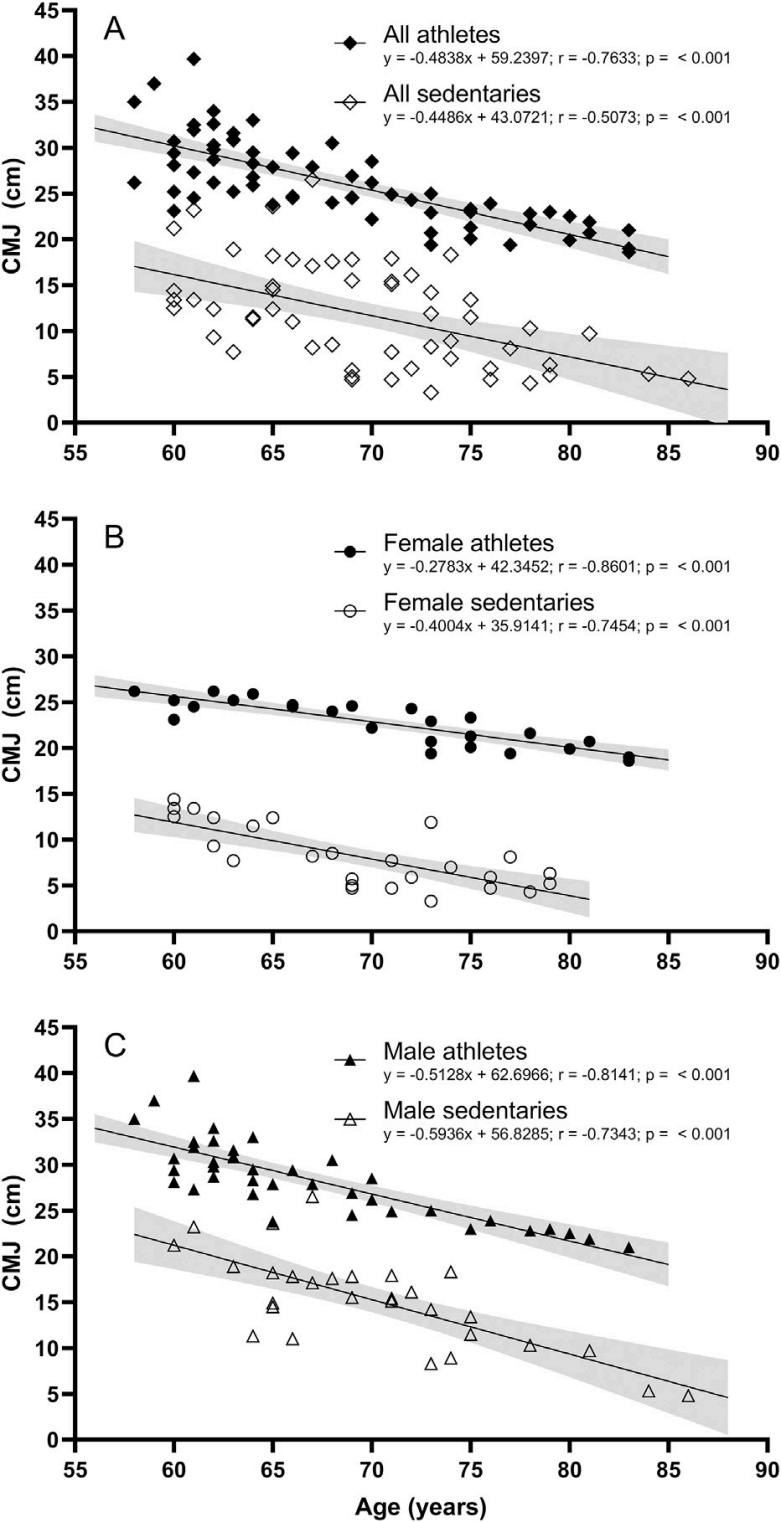
Correlations and 95% confidence intervals for CMJ performance and age in **(A)** All athletes and all sedentary participants; *p*-value for the slopes: 0.7473; **(B)** Female athletes and female sedentary participants; *p*-value for the slopes: 0.1203; and **(C)** Male athletes and male sedentary participants; *p*-value for the slopes: 0.4821.

Linear regression analysis showed a significant inverse correlation between age and jump height in all groups, with Pearson correlations (r) ranging from 0.7343 to 0.8601. Figure 2 compares athletes and sedentaries regression lines, and slopes showed no significant differences between these two groups for both females (Figure 2B, *p* = 0.1203) and males (Figure 2C, *p* = 0.4821).

**FIGURE 3:**
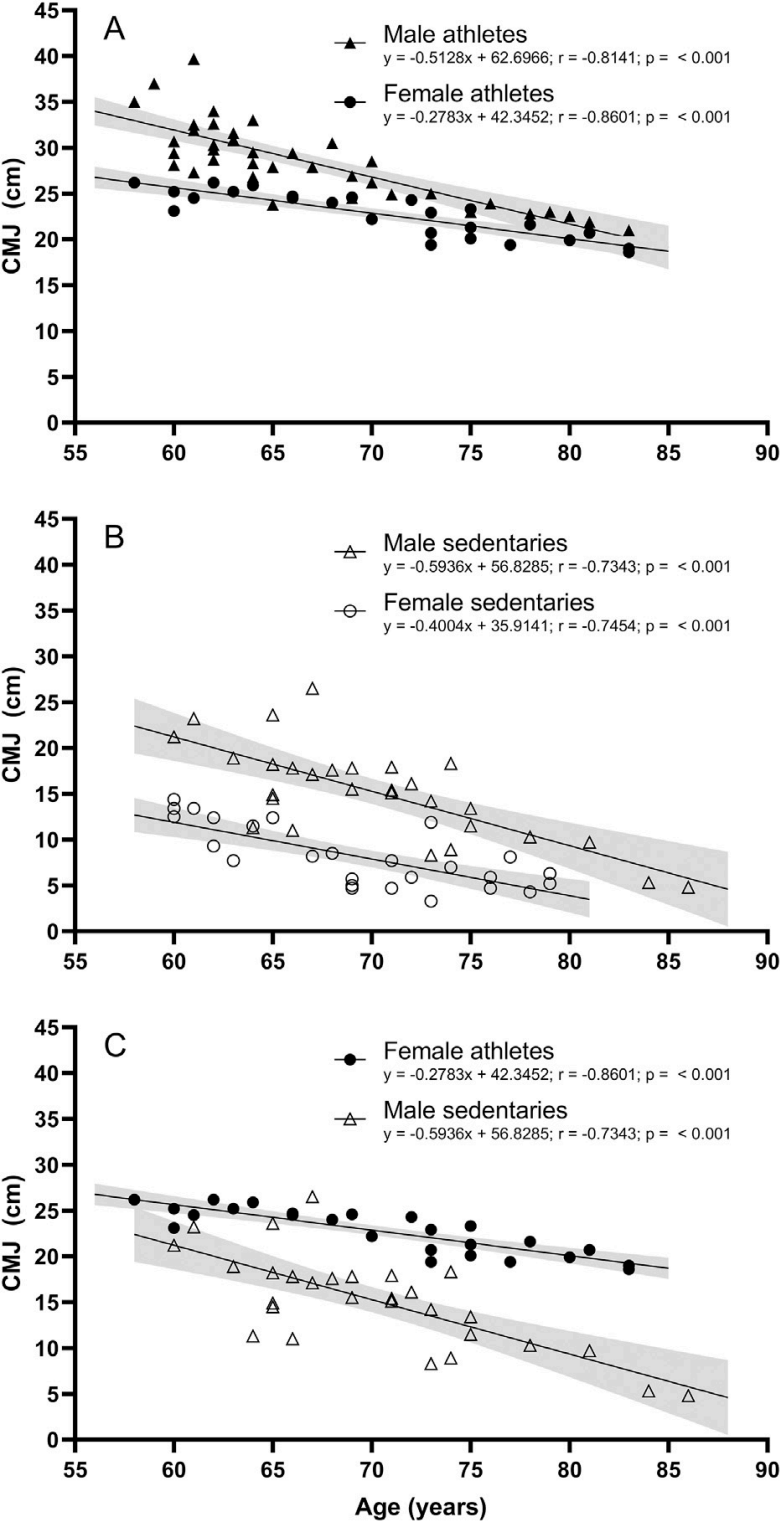
Correlations and 95% confidence intervals for CMJ performance and age in **(A)** Male athletes and female athletes; *p*-value for the slopes: 0.0033; **(B)** Male sedentaries and female sedentary participants; *p*-value for the slopes: 0.1455; and **(C)** Female athletes and male sedentary participants; *p*-value for the slopes: 0.0064.

Figure 3 compares males and females regression lines, both for athletes (Figure 3A) and for sedentaries (Figure 3B). Slopes were significantly different for male and female athletes (Figure 3A, *p* = 0.0033), but not for male and female sedentaries (Figure 3B, *p* = 0.1455). Female athletes had a different slope than male sedentaries (Figure 3C, *p* = 0.0064).

**FIGURE 4:**
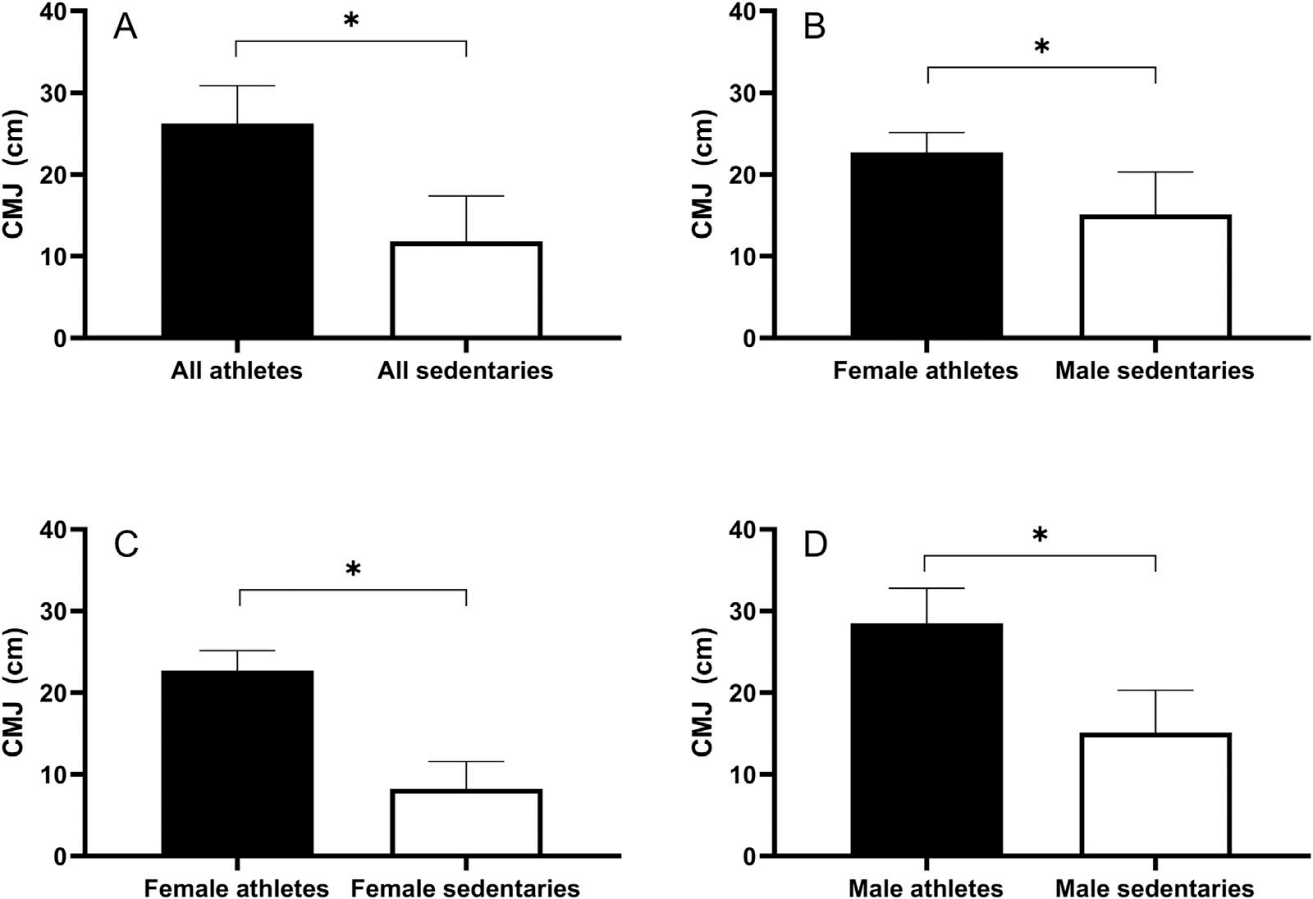
Average values (+SD) for CMJ performances for different gender and track and field training groups. In **(A)** all athletes vs all sedentary participants; **(B)** female athletes vs male sedentary participants; **(C)** female athletes vs female sedentary participants; **(D)** male athletes vs male sedentary participants. * = *p* < 0.01.

Figure 4 shows the average values of CMJ heights between athletes and sedentaries. The ANCOVA showed that mean CMJ heights were higher for the athletes than for the sedentaries in all groups (Figure 4). Regardless of gender, there were significant differences in the CMJ performances, with athletes showing higher values than sedentaries (26.2 ± 4.6 cm vs. 11.8 ± 5.6 cm; *p* < 0.01; Figure 4A). The *post hoc* analysis showed that the male athletes jumped significantly higher than their sedentary counterparts (28.5 ± 4.3 cm vs. 15.1 ± 5.2 cm; *p* < 0.01; Figure 4D) and the same was true for females (22.7 ± 2.5 cm vs. 8.2 ± 3.3 cm; *p* < 0.01; Figure 4C). Moreover, the female athletes jumped significantly higher than sedentary males (22.7 ± 2.5 cm vs. 15.1 ± 5.2 cm; *p* < 0.01; Figure 4B).

Additionaly, the CMJ heights of male athletes were significantly higher than those of female athletes (*p* < 0.01), and the same result was obtained for sedentary participants, where males jumped higher than females (*p* < 0.01).

## 4 Discussion

The main result of this study was that participating in sports activities such as track and field events significantly mitigates the effects of aging in the lower limb strength and functionality in individuals over 55, for both men and women. The ANCOVA results showed the superior performance of athletes over sedentary individuals in CMJ heights across all groups, regardless of gender (Figure 4). This is primarily because track and field events practiced by our master athletes require substantial lower body strength, and consistent training in these sports helps maintain high CMJ levels. This highlights the critical role of regular physical activity in preserving muscle functionality and explosive power of the lower limb extensors, which are essential for maintaining independence and functional capacity in older adults (Alvero-Cruz et al., 2021).

The observed decline in jump height aligns with the natural decay in explosive power of the lower limbs with advancing age, consistent with previous research showing similar coefficients of the regression lines for jump height decline among older adults (Alvero-Cruz et al., 2021). The CMJ is characterized by a high rate of force development, necessitating substantial motor unit recruitment. Previous research (Bosco and Komi, 1979; Bosco et al., 1983) indicates a positive correlation between jump height and the proportion of fast-twitch muscle fibers. However, with advancing age, muscle remodeling processes result in a transition from fast-twitch to slow-twitch fibers (Faulkner et al., 2007), impairing the elderly’s ability to execute movements that require a high rate of force development (Gillon et al., 2018). Consequently, the observed decline in CMJ height in aging can be partially attributed to the reduction in fast-twitch fibers of lower limb muscles. The slopes of jump heights were similar between athletes and sedentary individuals for both genders (Figure 2), suggesting that while track and field training enhances jump height, it does not alter the rate of decline in explosive power with age. This indicates that the benefits of track and field training are more pronounced in maintaining higher levels of explosive power rather than slowing its decline. Interestingly, when expressed as an annual percentage loss, the data suggest an alternative interpretation: male and female athletes experience smaller declines in CMJ performance (1.56% decline or −0.51 cm per year and 1.06% decline or −0.28 cm per year, respectively) compared to their sedentary counterparts (2.65% decline or −0.59 cm per year and 3.16% decline or −0.39 cm per year, respectively). This apparent difference is due to the higher initial strength levels in athletes. As a result, for the same absolute loss in centimeters, athletes experience a smaller percentage decline each year.

A study by Grassi et al. (1991) investigated the rate of age-related deterioration of maximal muscle power in two groups of power- or endurance-trained master athletes (n = 115; age range: 40–78 years). Two groups of young athletes (n = 20; 17–26 years) and healthy untrained subjects (n = 37; 22–67 years) were also tested for comparison. The results indicated a progressive reduction in muscle power after age 45, with peak power decreasing linearly as a function of age. By age 75, peak power was about 50% of the value measured at age 20, corresponding to a reduction of about 1% per year. Despite the data from the present study spans only from 58 to 86 years, this rate of decline is very similar to what we observed in our athletes. A similar result was found by Runge et al. (2004), in a physically active population, which showed a reduction of >50% in CMJ peak power between the ages of 20 and 80.

Gender comparisons revealed notable differences. As expected, male athletes had significantly higher CMJ heights than female athletes, and the same trend was observed among sedentary individuals. Among athletes, males and females exhibited significantly different slopes (Figure 3A), which can be attributed to the difference in muscle fiber composition between genders. Females generally have a higher proportion of slow-twitch muscle fibers (Nuzzo, 2024), which are more resistant to fatigue and better suited for endurance activities. This could result in a more gradual decline in explosive power compared to males, who typically have a higher proportion of fast-twitch muscle fibers that are more prone to age-related atrophy. These physiological differences underscore the importance of tailored training programs that consider gender-specific responses to exercise. However, this difference in slope was not observed among sedentary individuals (Figure 3B), suggesting that physical activity may amplify inherent gender differences in muscle performance (Alvero-Cruz et al., 2021). Additionally, female athletes showed a higher jumping capacity compared to male sedentaries (Figure 4B) and a shallower decline rate (Figure 3C), further emphasizing the influence of track and field training on maintaining muscle functionality.

Furthermore, low muscle power and strength in older adults have significant implications for fall risk. Reduced lower limb strength and power impair balance and mobility, increasing the likelihood of falls (Adams et al., 2023), which is a leading cause of injury and loss of independence in this population. Therefore, maintaining muscle strength and power through regular physical activities, such as track and field training, is crucial for reducing fall risk and promoting independence among older adults.

This study has some limitations. While the CMJ test is a valuable measure of lower limb strength and power, it may not be suitable for all older adults, particularly those with frailty or knee issues. Exercises focusing on flexibility, balance, aerobic, and resistance training might be more suitable to optimize the health of older and frail sedentary individuals (Theou et al., 2011). In contrast, for master athletes, jumping exercises might help maintain muscular strength, power and endurance, resulting in greater functional reserves for daily activities (Alvero-Cruz et al., 2021). Therefore, the aim of this study is not to recommend the CMJ as a general assessment tool for older populations, where more widespread tests like the Chair Stand Test might be more appropriate, but to use it to evaluate the influence of sporting activity and gender on lower limb strength decline.

Another limitation of this study is that despite all participants were instructed to jump with a knee angle of approximately 90°, this ability diminished with age. Six sedentary participants (10.7%) were unable to meet this criterion and instead utilized their preferred knee angle based on their lower limb strength and capacity, likely reaching their maximal jump height. It is improbable that these participants would have achieved greater jump heights with increased knee flexion, as their lower limb strength was insufficient to support such a movement. Consequently, the CMJs performed in our study exhibit high ecological validity, as they accurately reflect the natural adaptations and limitations of the participants’ physical capabilities.

In conclusion, this study demonstrates the significant benefits of track and field training in enhancing and maintaining lower limb explosive power in older adults. While the rate of decline in muscle performance with age remains consistent, engaging in regular sports activities during early, adult and older age can significantly elevate the baseline level of physical functionality, thereby promoting greater independence and quality of life during older age. Future research should explore the underlying mechanisms driving these gender differences and the long-term effects of various types of physical activity on muscle functionality in older adults.

## Data Availability

The raw data supporting the conclusions of this article will be made available by the authors, without undue reservation.
